# PDE5 inhibition eliminates cancer stem cells via induction of PKA signaling

**DOI:** 10.1038/s41419-017-0202-5

**Published:** 2018-02-07

**Authors:** Saskia Klutzny, Anna Anurin, Barbara Nicke, Joseph L. Regan, Martin Lange, Luise Schulze, Karsten Parczyk, Patrick Steigemann

**Affiliations:** 1 Bayer AG, Drug Discovery, 13353 Berlin, Germany; 2 Institute for Biotechnology, Bioanalytics, Technical University Berlin, 13355 Berlin, Germany

## Abstract

Cancer stem cells (CSCs) are involved in metastasis and resistance development, thus affecting anticancer therapy efficacy. The underlying pathways required for CSC maintenance and survival are not fully understood and only a limited number of treatment strategies to specifically target CSCs have been identified. To identify novel CSC targeting compounds, we here set-up an aldehyde dehydrogenase (ALDH)-based phenotypic screening system that allows for an automated and standardized identification of CSCs. By staining cancer cells for ALDH activity and applying high-content-based single-cell population analysis, the proportion of a potential CSC subpopulation with significantly higher ALDH activity (ALDH_high_) can be quantified in a heterogeneous cell population. We confirmed high ALDH activity as surrogate marker for the CSC subpopulation *in vitro* and validated Wnt signaling as an essential factor for the maintenance of CSCs in SUM149 breast cancer cells. In a small molecule screen, we identified phosphodiesterase type 5 (PDE5) inhibition as potential strategy to target CSC maintenance and survival in multiple cancer cell lines. CSC elimination by PDE5 inhibition was not dependent on PKG signaling, and we suggest a novel mechanism in which PDE5 inhibition leads to elevated cGMP levels that stimulate cAMP/PKA signaling to eliminate CSCs.

## Introduction

Metastasis and resistance development to chemotherapy and radiation are still a major obstacle in cancer treatment and pose a life-threatening condition for patients^1^. A restricted subset of tumor cells with self-renewing and differentiation properties similar to that of normal stem cells might be the reason for treatment failure and tumor reoccurrence. Those stem-like tumor cells have been termed cancer stem cells (CSCs) or tumor-initiating cells^2^. Targeting pathways responsible for CSC maintenance and survival in combination with drugs targeting the general tumor bulk could be a promising strategy to improve future clinical studies and patient outcome^3–5^.

However, identifying novel CSC-specific drugs by standard high-throughput assays, e.g., using cell viability as readout, is difficult as CSCs comprise only a small proportion of the cancer cell population^6^. So far, the main screening strategies have largely been based on genetic approaches including, for example, CSC enrichment by RNAi-based de novo CSC generation^6^, as well as coupling reporter genes to CSC-specific promoter sequences^7^. However, those approaches have been limited by their requirement for artificial CSC enrichment or by their limitation to known targets. Therefore, it was our aim to establish a versatile screening system that enables direct CSC quantification for the identification of potential novel targets and compounds that specifically target the CSC subpopulation.

CSCs can be characterized using a combination of specific markers^4^. Among those, the intracellular marker aldehyde dehydrogenase (ALDH) has increasingly been associated with the CSC phenotype in different solid tumor types^8–11^. The presence of cancer cells with enhanced ALDH activity in tumors correlates with increased tumorigenesis, poor prognosis, and increased metastasis^11–16^. ALDH activity can be measured using a fluorescent ALDH substrate that is trapped inside ALDH-positive cells (ALDH_high_ cells). ALDH_high_ cancer cells display significant higher tumor-initiating capacity as Aldefluor-negative cells *in vivo*^11,13^.

So far, Aldefluor has been applied for analyzing cells in flow cytometry, which, however, is not easily adjusted to high throughput and is often characterized by high experimental variations and lacking standardization^17^. Therefore, we here evaluated ALDH enzyme activity as a marker for CSCs in a phenotypic screening system and report for the first time a small molecule screen for CSC inhibitors using high-content-based single-cell population analysis. In addition to inhibitors of the canonical Wnt pathway, we identified four novel compounds that significantly affect the maintenance of ALDH_high_ CSCs in SUM149 breast cancer cells, including the cyclic guanine monophosphate (cGMP)-specific phosphodiesterase type 5 (PDE5) inhibitor MY5445. We provide data suggesting PDE5 as an important target in CSC maintenance in various cancer cell lines and propose PDE5 inhibition as potential treatment strategy in CSC-driven tumors. Moreover, we identified a novel mechanism which suggests that elevated cGMP levels by PDE5 inhibition act to stimulate cyclic adenosine monophosphate (cAMP)/protein kinase A (PKA) signaling, which in turn leads to the elimination of the CSC subpopulation.

## Results

### Set-up of a high-content phenotypic screening system to identify ALDH_high_ CSCs

**Fig. 1:**
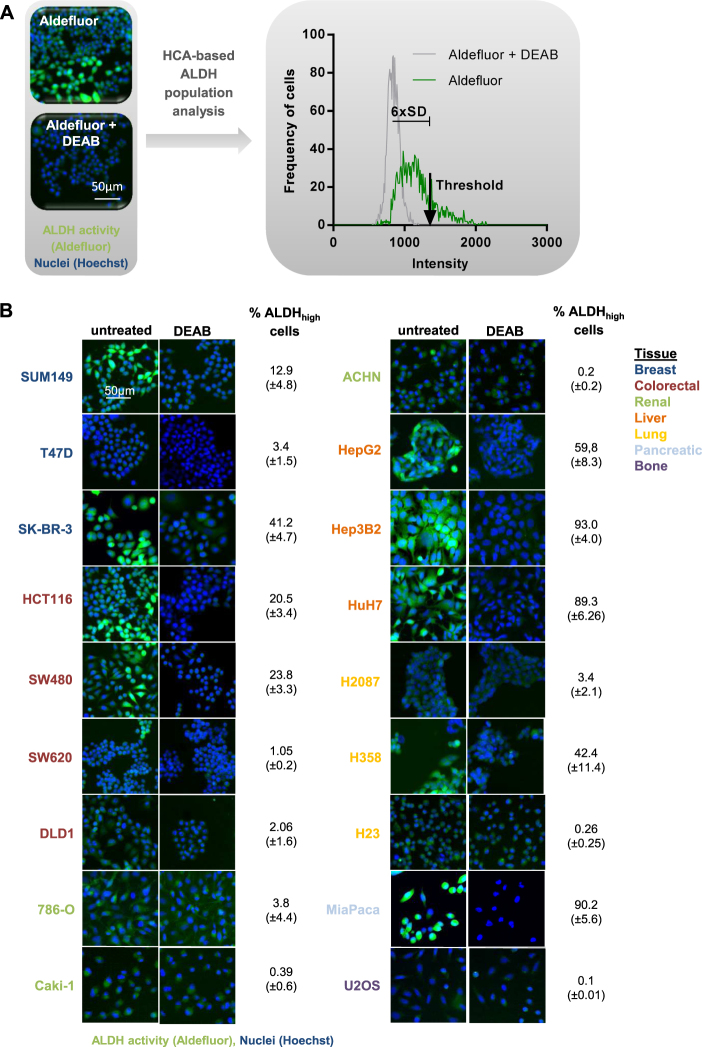
Set-up of a high-content phenotypic screening system to identify ALDH_high_ cancer stem cells (CSCs). **a** High-content-based ALDH population analysis for the identification of ALDH_high_ cells in a heterogeneous tumor cell line population. Cells are stained with the Aldefluor reagent and the nuclear stain Hoechst. A custom written image analysis algorithm is used to quantify Aldefluor staining intensities in every single cell of the entire cell population. A DEAB (ALDH inhibitor) treated cell population is used to set an intensity threshold at the average intensity + 6 × standard deviations (SD). The number of cells above this threshold is determined as percentage of the total population (ALDH_high_ cells). **b** High-content-based imaging and analysis of ALDH_high_ cells in different cell lines. Exemplary images of multiple experiments are shown (*n* ≥ 2). Scale bar 50 µm

To identify the proportion of ALDH_high_ cells in a heterogeneous cell population, we adapted the Aldefluor flow cytometry staining protocol to establish a high-throughput, microscopy-based cell population analysis assay. To quantify the proportion of the ALDH_high_ CSC subpopulation, the average Aldefluor background intensity in cells treated with the ALDH inhibitor diethylaminobenzaldehyde (DEAB) was determined and a cutoff was set at six standard deviations above this average (Fig. 1a). The number of cells above this intensity threshold was measured in the sample of interest and given as the proportion of ALDH_high_ cells. This allowed for a standardized and precise way to identify the ALDH_high_ subpopulation^17^. Using this novel high-content-based Aldefluor cell population analysis set-up, we analyzed various cell lines of different tissue origin for their proportion of ALDH_high_ cells (Fig. 1b). We found large variations in the proportion of ALDH_high_ cells not only between cell lines of the same tissue origin but also between cancer cells from different tissues. The frequency of ALDH_high_ cells in each cell line was in agreement with previously published flow cytometric data^11,18–23^. For all the following experiments, we concentrated on the breast cancer cell line SUM149, which demonstrated a consistent Aldefluor staining profile and has been well characterized in literature, both *in vitro* and *invivo*^11,13,24–26^.

### ALDH_high_ cancer cells demonstrate enhanced TFC and radiation resistance

3D cell culture models that monitor tumorsphere formation are a surrogate for *in vivo* models to measure the tumorigenic potential of cancer cells *in vitro*. Here we tested and adapted an anchorage-independent growth assay system to monitor tumorsphere formation to determine the tumorsphere-formation capacity (TFC) in a throughput-compatible 384-well format (described in detail in Materials and Methods).

**Fig. 2:**
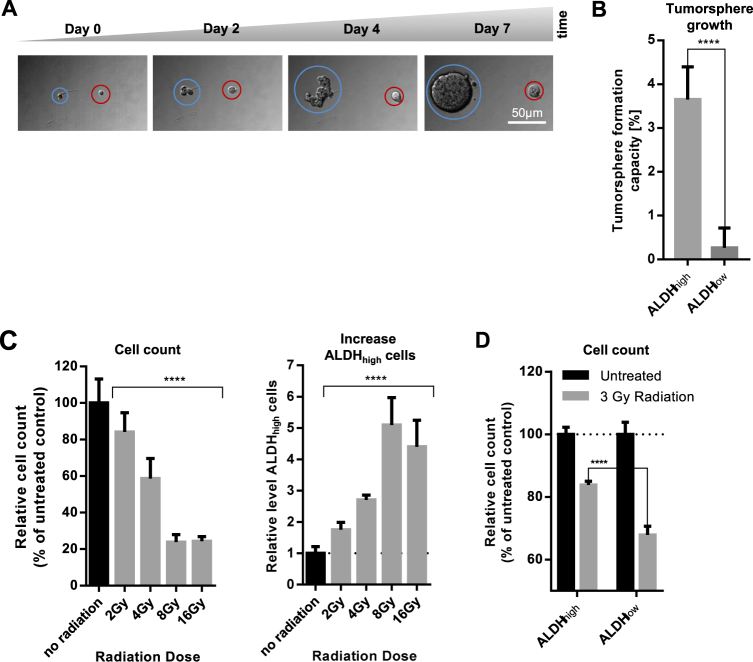
ALDH_high_ cancer cells demonstrate enhanced tumorsphere formation and resistance to radiation. **a** Monitoring tumorsphere formation from single cells. A single-cell suspension of SUM149 (100 cells per well) was plated in 384-well tumorsphere plates. Transmitted light images of the same site were acquired at multiple time points. Circled in red: cell showing no tumorsphere growth. Circled in blue: cell forming a tumorsphere. Scale bar 50 µm. **b** SUM149 cells were sorted into ALDH_high_ and ALDH_low_ cells by FACS. Two hundred cells of each population were plated in 384-well tumorsphere plates. After 7 days of growth, the number of tumorspheres was determined and normalized to the initial seeding cell number. Bars show mean with SD (*n* = 3). ****p-value < 0.0001. **c** SUM149 cells were plated in 384-well plates and irradiated with different doses. After 72 h, total cell numbers and the amount of ALDH_high_ cells were determined and normalized to no radiation control. DEAB was used as inhibitor staining control to set intensity threshold of Aldefluor staining. Bars show mean with SD (*n* = 3). ****p-value < 0.0001. **d** SUM149 cells were sorted by FACS into ALDH_high_ and ALDH_low_ cell populations and subsequently plated into 384-well plates, maintained in normal growth medium for 20 h, and subjected to radiation (3 Gy). After 72 h, cells were stained with the nuclear marker Hoechst and the dead cell stain SytoxGreen. Viable cells were quantified and normalized to the untreated sample (non-irradiated cells)

After Aldefluor staining the breast cancer cell line SUM149 shows a heterogeneous population of cells with different levels of ALDH activity (Fig. 1). Accordingly, SUM149 cells display different capacities for anchorage-independent growth (Fig. 2a and supplementary figure 2A). While some cells formed tumorspheres (circled in blue), others showed no growth (circled in red), indicating the existence of cells with different tumorigenic potential within this cell line. SUM149 cells were sorted into ALDH_high_ and ALDH_low_ cells by flow cytometry and tested for their tumor forming capacity. Indeed, ALDH_high_ cells showed a significantly higher capacity of tumorsphere formation than ALDH_low_ cells (Fig. 2b and supplementary figure 2B). Furthermore, we found that tumorsphere formation correlates with the presence of an ALDH_high_ subpopulation in other cell lines (Fig. 1b and supplementary figure 2C).

In addition to an enhanced tumor formation capacity CSCs have also been reported to be the source of radiation resistance^27,28^. Accordingly, irradiation of SUM149 led to a dose-dependent decrease in total cell number but at the same time to an increase of ALDH_high_ cells (Fig. 2c), indicating that ALDH_high_ cells are more resistant to radiation. Indeed, when isolating ALDH_high_ and ALDH_low_ cells by FACS and exposing them to irradiation, ALDH_high_ cells demonstrate an enhanced ability to survive radiation (Fig. 2d).

Taken together, these results demonstrate an enhanced TFC and radiation resistance of ALDH_high_ cells and validate ALDH activity as marker for CSCs in SUM149 cells.

### Wnt inhibition reduces the level of ALDH_high_ CSCs in SUM149 cells

**Fig. 3:**
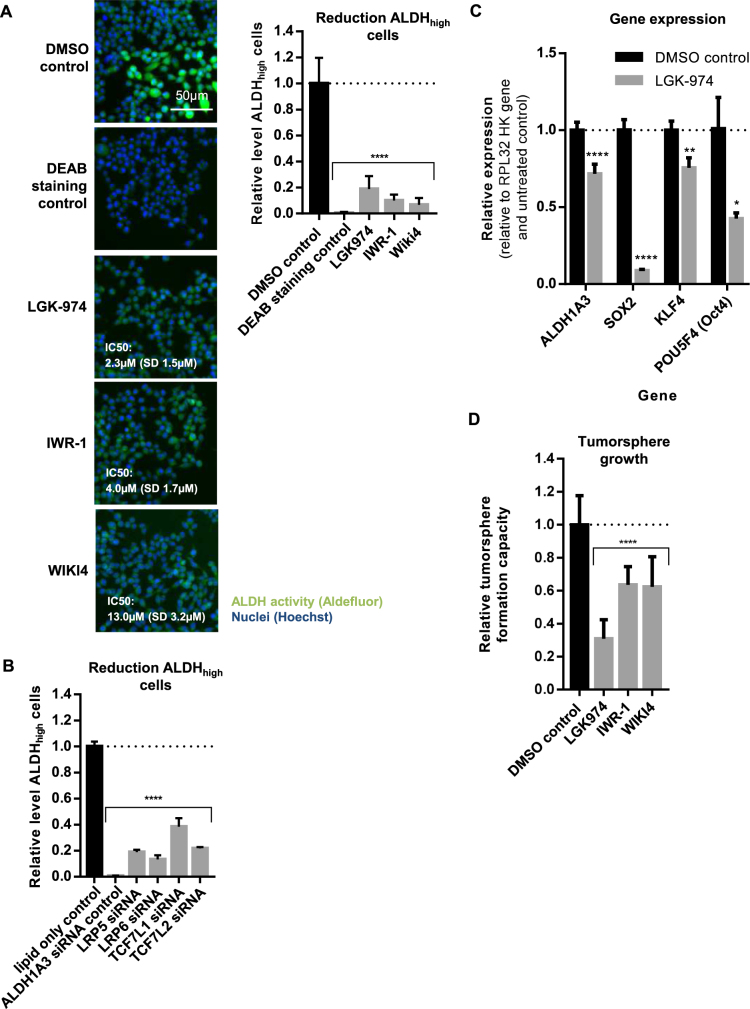
Wnt inhibition reduces the level of ALDH_high_ cells and the tumorsphere formation in SUM149 cells. **a** SUM149 cells were treated with either DMSO control or 10 µM Wnt inhibitor (LGK-974, IWR-1, or WIKI4). After 72 h, the amount of ALDH_high_ cells was determined and normalized to DMSO control. DEAB was used as inhibitor staining control to set intensity threshold of Aldefluor staining. Exemplary images of multiple experiments are shown (*n* ≥ 3). Scale bar 50 µm. IC50 values were determined in *n* ≥ 3 independent experiments. Bars in graph show mean with SD (*n* ≥ 3). ****p-value < 0.0001. **b** SUM149 cells were treated with either lipid only control or 10 nm siRNA. After 72 h, the amount of ALDH_high_ cells was determined and normalized to lipid only control. ALDH1A3 siRNA was used as inhibitor control to set intensity threshold of Aldefluor staining. Bars show mean with SD (*n* ≥ 3). ****p-value < 0.0001. **c** Gene expression analysis of stemness-associated marker genes by RT-PCR in SUM149 cells treated for 72 h with either DMSO control or 10 µM LGK-974. hRP-L32 was used as reference gene and relative expression levels were normalized to DMSO control. Bars show mean with SD (*n* = 3). ****p-value<0.0001, **p-value<0.01, *p-value<0.05** d** SUM149 cells were plated in 384-well tumorsphere plates and treated with either DMSO control or 10 µM Wnt inhibitor (LGK-974, IWR-1, or WIKI4). After 7 days of growth, the number of tumorspheres was determined and normalized to initial seeding cell numbers and DMSO control. Bars show mean with SD (*n* = 3). ****p-value < 0.0001

To elucidate the main mechanisms required for CSC maintenance in SUM149 cells we tested multiple inhibitors of the three most prominent CSC maintenance pathways^3,4,29,30^, Wnt, Notch and Hh, for their ability to reduce the ALDH_high_ CSC subpopulation (complete list see supplementary table 1). While inhibitors of Notch and Hh signaling pathway showed only minor or no reduction of ALDH_high_ cells in SUM149 after 72 h incubation, we identified three Wnt inhibitors (LGK-974 which targets porcupine and WIKI4 and IWR-1, which target Tankyrase/Axin) that showed a concentration dependent decrease of ALDH_high_ cells without affecting general cell viability (Fig. 3a). These findings were further validated by a focused siRNA screen covering different nodes of all three pathways (see supplementary table 1). Similar to the tested small molecule inhibitors, mainly siRNA against targets of the Wnt signaling pathway led to a significant reduction of ALDH_high_ SUM149 cells after 72 h incubation (Fig. 3b and supplementary table 1).

To further investigate the effect of Wnt inhibition on the maintenance of CSCs in SUM149, we performed reverse transcriptase-PCR (RT-PCR) analysis for different stemness-associated marker genes. Indeed, as expected, treatment with the porcupine inhibitor LGK-974 decreased the expression of ALDH1A3 and also reduced the expression level of CSC-associated genes like *SOX2*, *KLF4*, and *POU5F4 (Oct4)* (Fig. 3c)^31–34^. Additionally, we functionally evaluated the effect of Wnt inhibition on tumorsphere formation *in vitro*. All three inhibitors significantly reduced the TFC of SUM149 after 7 days incubation (Fig. 3d).

In summary, these data show that active Wnt signaling is an essential factor for the maintenance of CSCs in SUM149. Moreover, they provide proof of principle for the identification of CSC maintenance inhibitors using the established high-content-based Aldefluor and TFC assays.

### ALDH activity is not required for CSC maintenance in SUM149 cells

**Fig. 4:**
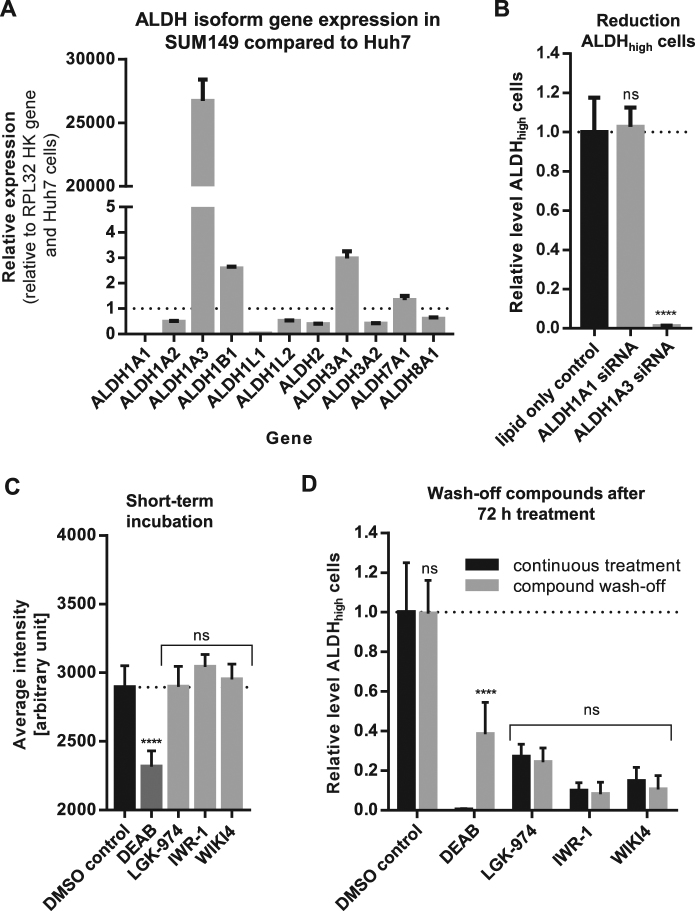
ALDH activity is not required for CSC maintenance in SUM149 cells. **a** Gene expression analysis of 11 common ALDH isoforms by RT-PCR in SUM149 compared to Huh7. Ct values were normalized with the internal control RPL32. Expression of ALDH isoforms in SUM149 was compared to Huh7 and relative gene expression levels were determined. Bars show mean with SD (*n* = 3). **b** SUM149 cells were treated with either lipid only control or 10 nm siRNA. After 72 h, the amount of ALDH_high_ cells was determined and normalized to lipid only control. DEAB was used as inhibitor staining control to set intensity threshold of Aldefluor staining. Bars show mean with SD (*n* = 3). ****p-value < 0.0001, ns—not significant. **c** SUM149 cells were treated for 2 h with either DMSO control or 10 µM compound in Aldefluor assay buffer. After 2 h incubation, the average Aldefluor staining intensity was determined. Bars show mean with SD (*n* = 3). ****p-value < 0.0001, ns—not significant. **d** SUM149 cells were treated for 72 h with either DMSO control or 10 µM compound. After 72 h, medium was changed to wash-off compounds and cells were incubated for another 2 h in Aldefluor assay buffer (without compound). The amount of ALDH_high_ cells was determined and normalized to DMSO control. Freshly added DEAB was used as inhibitor staining control to set intensity threshold of Aldefluor staining. Bars show mean with SD (*n* = 3). Significance was calculated for each compound comparing compound wash-off with no wash-off (continuous treatment). ****p-value < 0.0001, ns—not significant

Increased ALDH activity has not only been proposed as CSC marker in different tumor types and cell lines but also as therapeutic cancer target, in particular the two ALDH isoforms ALDH1A1 and ALDH1A3^35–37^. To identify the main isoform responsible for ALDH activity in SUM149 cells, we profiled the expression of the most common ALDH isoforms by RT-PCR and compared these to the liver cancer cell line Huh7. While in Huh7 cells *ALDH1A1* showed the highest relative expression, *ALDH1A3* showed the highest expression in SUM149 (Fig. 4a). Therefore, we speculated that the CSC subpopulation in SUM149 cells could be marked by *ALDH1A3* expression. Indeed, siRNA against *ALDH1A3* completely prevented retention of the Aldefluor reagent in SUM149 cells, while *ALDH1A1* siRNA had no significant effect (Fig. 4b).

To evaluate ALDH, in particular ALDH1A3, as potential target in CSC maintenance, we tested the effect of multiple ALDH inhibitors with different isoform specificity^38^, including DEAB (pan-inhibitor, most specific for ALDH1 isoform^39^), Daidzin (ALDH2 inhibitor^40^), Gossypol (pan-inhibitor, most specific for ALDH3 isoform^41^), and Disulfiram (pan-inhibitor, most specific for ALDH1A1^42^) on SUM149 breast cancer cells (data not shown). In accordance with the ALDH isoform expression level in SUM149, only DEAB strongly reduced the level of ALDH_high_ cells. Importantly, DEAB was already active after a short incubation time of 2 h, while Wnt pathway inhibitors required at least 48 h incubation to reduce the level of ALDH_high_ cells in SUM149 (Fig. 4c).

Based on these results, we hypothesized that targeting the maintenance of ALDH_high_ CSC subpopulation, e.g., via Wnt inhibition, requires a longer period of time in order to induce CSC-specific cell death or differentiation. In contrast, inhibiting ALDH enzyme activity via DEAB might only interfere with the transformation of the Aldefluor substrate and not the maintenance of CSCs. Supporting this, we observed a reappearance of ALDH_high_ cells after compound wash-off only in wells that had been treated for 72 h with DEAB but not in cells treated with a Wnt inhibitor (Fig. 4d). Moreover, DEAB also did not affect the tumorsphere formation in SUM149 in anchorage-independent growth conditions (reduction in TFC compared to DMSO control: −13.4% (SD ± 6.2%)).

Taken together, we conclude that ALDH inhibition only masks the Aldefluor signal but is not required for CSC maintenance in SUM149 cells. Hence, ALDH inhibition does not seem to be a suitable strategy for functionally targeting SUM149 CSCs.

### Screen for the identification of novel CSC inhibitors

To identify potential novel CSC inhibitors and target pathways, we performed a high-content-based Aldefluor screen in SUM149 on a drug library of known bioactive substances and Food and Drug Administration (FDA)-approved drugs (1108 compounds, *n* = 4 per compound). Toxic and antiproliferative substances that reduced the cell number by more than 50% compared to the solvent control (DMSO) were excluded. Compounds that reduced the level of ALDH_high_ cells by >25% were nominated as hits. Ten compounds showed a concentration-dependent reduction of ALDH_high_ CSCs (see Table 1). All confirmed hits, as well as the three Wnt-pathway inhibitors, were also profiled in HCT116 colon cancer cells and showed similar effects (supplementary table 2).

**Table 1:** Reduction of ALDH_high_ cells and tumorsphere formation capacity (TFC) in SUM149 cells

ALDH assay: SUM149 cells were treated for 72 h (IC50 generation) or 2 h (short-term incubation, compound concentration 10 µM). Nuclei were stained with Hoechst and ALDH activity visualized by Aldefluor staining. The amount of ALDH_high_ cells was determined and normalized to DMSO control. DEAB was used as inhibitor staining control to set intensity threshold of Aldefluor staining. Values were determined in ≥3 independent experiments. SD of IC50 values in brackets. Colored gradation of IC50 values: from dark green = best IC50 to white = weakest IC50. For 2 h incubation, SD and significance compared to DMSO control in brackets (****p-value < 0.0001, ns = not significant). Red = false-positive fast acting compound

TFC assay: SUM149 were plated as single-cell suspension (200 cells/well) in 384-well tumorsphere plates and treated with compounds (10 µM) or DMSO control. After 7 days of growth, the number of tumorspheres was determined and normalized to DMSO control. Reduction of TCF was determined in *n* = 3 indepe*n*dent experiments. SD and significance compared to DMSO control in brackets (****p-value < 0.0001, *p-value between 0.01 and 0.05, ns = not significant). Significant reduction of TFC in colored in green

Next, hits were tested for their inhibitory effect on tumorsphere formation of SUM149 cells. For this, compounds were used at concentrations that showed no toxicity but were high enough to significantly decrease the level of ALDH_high_ cells. The reduction of TFC (relative to the DMSO control) for all hit compounds is summarized in Table 1. Only four compounds significantly (*p*-value < 0.0001) reduced tumorsphere formation by >50%.

We speculated that, similar to DEAB, ALDH inhibitors should have no effect on TFC and lead to rapid inhibition of ALDH activity. To identify ALDH inhibitors in the hitlist, we incubated SUM149 cells in Aldefluor assay buffer together with the hit compounds for 2 h and measured the proportion of ALDH_high_ cells (see Table 1). Five compounds showed a strong reduction of ALDH_high_ cells, similar to that of the known ALDH inhibitor DEAB (94% (±2.7%) reduction). Furthermore, we observed a reappearance of ALDH_high_ cells after compound wash-off only in wells treated with these five fast acting hits (supplementary figure 3).

Taken together, from 1108 screened compounds from a drug library of known bioactive substances and FDA-approved drugs, 10 compounds could be identified that reduce the level of ALDH_high_ cells in SUM149 cells. However, 5 of the 10 identified compounds could be identified as potential false positives, as they lead to rapid ALDH inhibition and had no functional effect on CSCs. Therefore, we excluded these as false positives from the hit list.

### PDE5 inhibition reduces stem-like ALDH_high_ cells

Similar to Wnt pathway inhibitors, four of the remaining hits showed characteristics of a CSC maintenance inhibitor (reduction ALDH_high_ cells and decrease in TFC). One of the most potent compounds of these is the PDE5 inhibitor MY5445 (see Table 1). In addition to SUM149 breast cancer cells, MY5445 also significantly reduced the level of ALDH_high_ cells in HCT116 colon cancer cells and H358 lung cancer cells (supplementary figure 3 B), suggesting PDE5 as an important target for CSC maintenance in different tumor cell types. Indeed, Liu et al. also demonstrated an essential role of PDE5 in the survival of prostate CSCs^43^.

**Fig. 5:**
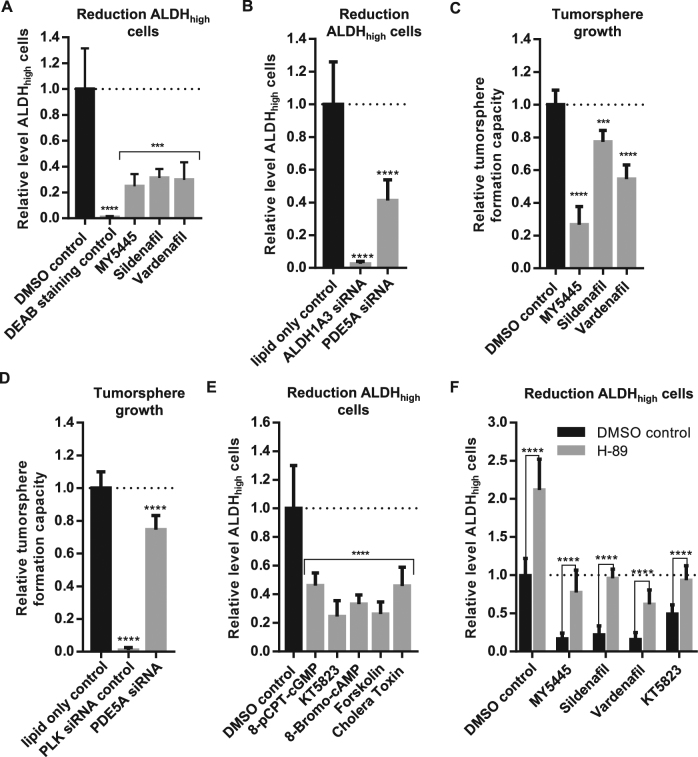
PDE5 inhibition reduces the level of ALDH_high_ cells and the tumorsphere formation in SUM149 cells. **a**, **b** SUM149 cells were treated with either DMSO control or 10 µM PDE5 inhibitor. For gene knockdown, SUM149 were treated with either lipid only control or 10 nm siRNA (ALDH1A3 or PDE5A). After 72 h, the amount of ALDH_high_ cells was determined and normalized to **a** DMSO control or **b** lipid only control. DEAB (**a**) or ALDH1A3 siRNA (**b**) was used as inhibitor staining control to set intensity threshold of Aldefluor staining. Bars show mean with SD (*n* = 3). ****p-value < 0.0001, ***p-value < 0.001. **c**, **d** SUM149 cells were treated with either DMSO control or 10 µM PDE5 inhibitor and for gene knockdown with either lipid only control or 10 nm siRNA (PLK control or PDE5A) under anchorage-independent growth conditions (384-well tumorsphere plates). After 7 days of growth, the number of tumorspheres was determined and normalized to initial seeding cell numbers and **c** DMSO or **d** lipid only control. Bars show mean with SD (*n* = 3). ****p-value < 0.0001, ***p-value<0.001. **e** SUM149 cells were treated for 72 h with either DMSO control, 250 µM 8-pCPT-cGMP, 10 µM KT5823 (PKG inhibitor), 25 µM 8-Bromo-cAMP, 10 µM Forskolin, or 5 µg/ml Cholera toxin (cAMP activator). After 72 h, the amount of ALDH_high_ cells was determined and normalized to DMSO control. DEAB was used as inhibitor staining control to set intensity threshold of Aldefluor staining. Bars show mean with SD (*n* = 3). ****p-value < 0.0001. **f** SUM149 cells were co-treated with 5 µM H-89 (PKA inhibitor) and either 10 µM PDE5 inhibitor or 10 µM KT5823 (PKG inhibitor). After 72 h, the amount of ALDH_high_ cells was determined and normalized to DMSO control. DEAB was used as inhibitor staining control to set intensity threshold of Aldefluor staining. Bars show mean with SD (*n* = 3). ****p-value < 0.0001)

To further validate PDE5 inhibition as a potential CSC maintenance target, we tested two other small molecule inhibitors of PDE5 on SUM149 cells. Both clinically approved PDE5 inhibitors Sildenafil and Vardenafil also reduced the level of ALDH_high_ cells (see Fig. 5a). Supporting these findings, *PDE5A* gene knockdown by siRNA also showed a significant reduction of ALDH_high_ cells (Fig. 5b). Moreover, PDE5 inhibition by specific small molecule inhibitors or gene knockdown of PDE5A by siRNA reduced the tumorsphere formation of SUM149 cells *in vitro* (see Fig. 5c, d and supplemental Fig. 4).

PDE5 controls the degradation of the second messenger cGMP, an activator of protein kinase G (PKG)^44^. Therefore, we speculated that treating cells with a cGMP analog should sufficiently increase cGMP level to mimic the effect of PDE5 inhibition. Indeed, similar to MY5445, the cGMP analog 8-(4-chlorophenylthio)-guanosine 3′,5′-cyclic monophosphate (8-pCPT-cGMP) reduced the level of ALDH_high_ cells in SUM149 (Fig. 5e). Based on these results, we hypothesized, that cGMP-dependent PKG activity could regulate the maintenance of CSCs. Consequently, PKG inhibition should reverse the effect and prevent the reduction of ALDH_high_ CSCs^43^. However, on the contrary, PKG inhibition by KT5823 significantly reduced the level of ALDH_high_ cells and tumorspheres (Fig. 5e and supplemental Fig. 5).

In addition to activating PKG, cGMP has also been shown to inhibit the degradation of cAMP by other PDE family members^45^. Accordingly, we also find a reduction of ALDH_high_ cells when increasing cellular cAMP levels by Forskolin or Cholera toxin treatment or by exogenous addition of the cAMP analog 8-Bromo-cAMP (Fig. 5e), suggesting a role of cAMP signaling in CSC maintenance. Indeed, inhibition of the cAMP-dependent PKA by H-89 increased the level of ALDH_high_ cells, rescued the CSC-decreasing effects of PDE5 or PKG inhibition in SUM149, and had no effects on tumorsphere formation (Fig. 5f and supplemental Fig. 5).

**Fig. 6:**
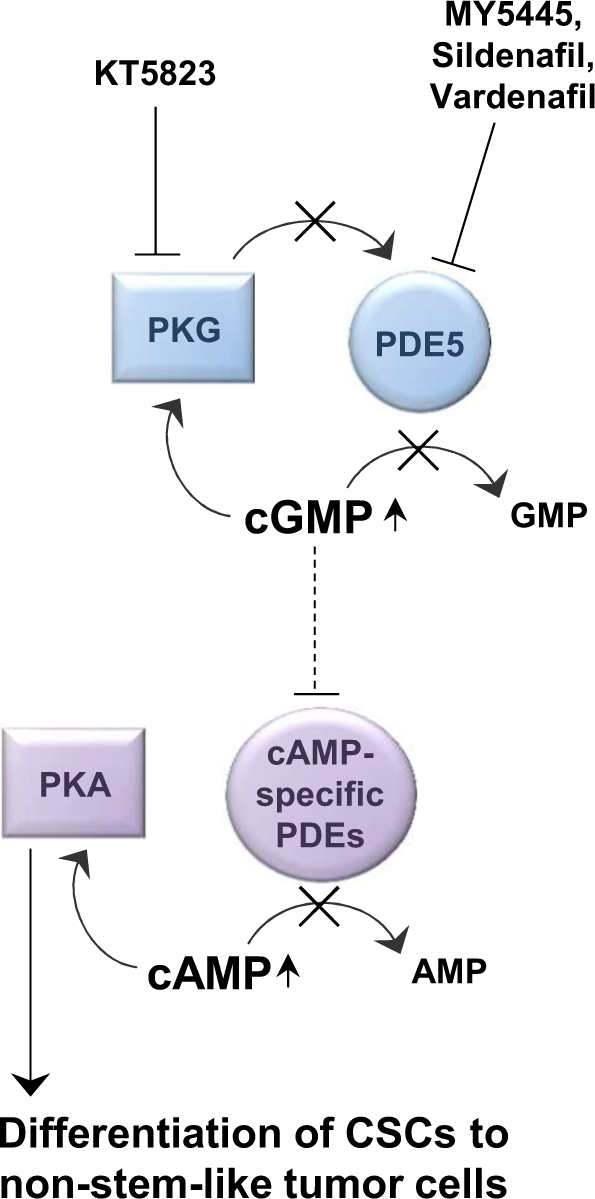
Potential model for targeting the maintenance of CSCs: interaction of PDE5 with cGMP/PKG and cAMP/PKA signaling pathways modulates the maintenance of CSCs. PDE5 or PKG inhibition increases cGMP levels, which stimulate the activity of other PDE family members to block the degradation of cAMP. Increased cAMP levels in turn activate PKA and induce the differentiation of CSCs to non-stem-like tumor cells

Summarizing, these results suggest that PDE5 inhibition eliminates CSCs via induction of PKA signaling (Fig. 6) and thereby represents a potential treatment opportunity to enhanced chemotherapy and radiation therapy efficacy in CSC-driven tumors.

## Discussion

By using ALDH activity as stemness marker, we set up a screening system that allows for an automated and standardized identification of CSC-targeting compounds. This approach was validated by the identification of Wnt pathway inhibitors as CSC-targeting compounds. Wnt signaling influences the expression of hundreds of diverse genes and is involved in the maintenance of normal stem cells^46,47^. However, in past years, aberrant regulation of the Wnt pathway has increasingly been associated with various cancer types and closely correlates with increased tumor progression and resistance development. Moreover, it appears that Wnt signaling not only has a vital role for the function of normal stem cells but also for CSCs^3,30,46^. Indeed, by treatment with Wnt pathway inhibitors or siRNA-mediated gene knockdown we here confirmed the canonical Wnt pathway being required for CSC maintenance in SUM149 breast cancer cells. Therefore, inhibition of Wnt signaling could be a promising target strategy for cancer treatment. However, despite promising results in preclinical studies^46–48^, the development of clinical relevant therapies is hampered by the pivotal role of Wnt signaling in tissue development and self-renewal of normal stem cells that causes a challenging safety profile^47,49^.

Owing to the obstacles with current approaches like Wnt inhibition, there is a high need to identify novel targets and CSC-targeting drugs. Therefore, we here report for the first time a small molecule screen for CSC maintenance inhibitors using high-content-based single-cell population analysis of Aldefluor-stained cancer cells. We identify PDE5 inhibition as potential strategy to functionally target CSCs. In contrast to Wnt inhibitors, PDE5 inhibitors have long been used in the clinic to treat erectile dysfunction and show an acceptable side effect profile^51^. Additionally, increasing evidence suggests PDE5 also as an oncological target^52^. As PDE5 regulates the degradation of cGMP, a second messenger that controls cell growth and apoptosis, elevated PDE5 levels have been associated with tumorigenesis in multiple cancer types, such as colon, pancreatic, prostate, lung, or breast carcinoma^52^. Moreover, treatment with PDE5 inhibitors has been suggested to reduce the risk for prostate cancer^53^ and first preclinical studies in cancer models showed positive anticancer results^54–57^. Additionally, PDE5 has been associated with the maintenance of prostate CSCs^43^. However, the use of PDE5 inhibitors in cancer therapy remains controversial^58^.

We here provide further evidence that confirms PDE5 signaling as crucial factor for the maintenance of CSCs in multiple cancer cell lines and highlights the upregulation of cGMP via PDE5 inhibition as a promising strategy to target CSC-containing tumors. Importantly, our findings suggest that the CSC-targeting effect of PDE5 inhibition is not mediated by increased activation of PKG^43^ but by an alternative cGMP-dependent mechanism. Indeed, PKG inhibition does not increase the level of ALDH_high_ CSCs but phenocopies the effect of PDE5 inhibition, possibly by feedback inhibitory characteristics of PKG to balance cGMP levels^44,52^ (Fig. 6). Instead, we provide evidence that the accumulation of cGMP by PDE5 inhibition upregulates cAMP-dependent PKA activity, as the effects seen with PDE5 inhibitors can be rescued by co-inhibition of PKA. Moreover, elevated cAMP signaling in cells treated with a cAMP activator or cAMP analogs resulted in a reduction of CSCs. This indicates a pivotal role of cAMP/PKA signaling in CSC regulation.

High levels of cGMP have been shown to inhibit PDE1- and PDE3-mediated hydrolysis of cAMP^45^. Therefore, we speculate that elevated cGMP levels induce cAMP accumulation and subsequent activation of PKA signaling possibly via PDE1 or PDE3 inhibition. However, none of the tested PDE1 (8-MM-IBMX and Vinpocetine) or PDE3 (Milrinon und Amrinon) inhibitors showed similar effects as PDE5 inhibitors (data not shown) and therefore the exact link between cGMP and PKA signaling remains to be determined. Nevertheless, recent results^59^ confirm that PKA can induce the differentiation of breast CSCs. Based on these findings, we propose a model in which elevated cGMP levels block the degradation of cAMP possibly by modulating the activity of other PDE family members. Increased cAMP levels in turn activate PKA and induce the differentiation of CSCs (Fig. 6).

Concluding, we here provide novel insights into the interaction of PDE5 with cGMP/PKG and cAMP/PKA signaling pathways and propose a new mode of action for the involvement of PDE5 in the maintenance of CSCs. Moreover, our findings suggest PDE5 inhibition as a potential strategy to target CSC-containing tumors and to increase their susceptibility to conventional chemotherapeutic drugs and radiation therapy. Nevertheless, established 2D cultured cell lines are only a limited model for CSCs found in a real tumor. Therefore, additional work using fresh patient-derived cancer cells or animal models will be required to further evaluate this approach.

## Materials and methods

### Cell culture

All cell lines were obtained from American Type Culture Collection. SUM149, MiaPaca, 786-O, and Caki-1 cells were cultured in Dulbecco’s modified Eagle’s medium (DMEM) HAM's F12 (Gibco by Thermo Fisher, Waltham, MA, USA). HCT116, H358, T47D (+0.01 µg/ml Insulin), SW620, SW480, DLD1, HepG2, H2087, H23, and U2OS cells were cultured in RPMI1640 (Gibco by Thermo Fisher, Waltham, MA, USA). ACH, Hep3B2, and Huh7 cells were cultured in DMEM. SK-BR-3 cells were cultured in McCoy’s 5A: All media were supplemented with 10% fetal calf serum (PAA Laboratories by GE Healthcare, Little Chalfont, UK) and 1% Penicillin/Streptomycin (Sigma-Aldrich, St. Louis, MO, USA). Cells were maintained at 37 °C in a 5% CO_2_ and 95% air incubator.

### High-content-based Aldefluor assay and single-cell population analysis

To identify the proportion of CSCs with a high ALDH activity in a heterogeneous cell population, cells were seeded in 40 µl medium into 384-well plates and incubated overnight. After optional compound treatment, medium was removed and cells were washed once with Dulbecco’s Phosphate Buffered Saline, before the activated Aldefluor reagent diluted in assay-specific buffer (1:400, STEMCELL Technologies, Vancouver, Canada) was added to the cells together with the nuclear stain Hoechst 33,342 (1:5000, Life Technologies by Thermo Fisher, Waltham, MA, USA). DEAB, an ALDH inhibitor, was used as inhibitor staining control (10 µM, STEMCELL Technologies, Vancouver, Canada). Cells were incubated for a minimum of 45 min at 37 °C before automated imaging using an Opera confocal spinning disc microscope system (PerkinElmer, Waltham, MA, USA) with a 10× air objective. The fluorescent signal is stable over multiple hours in living cells (see supplementary figure 1).

Quantification of total cell number and proportion of ALDH_high_ cells was done with the MetaXpress software (Molecular Devices, Sunnyvale, CA, USA) using custom written single-cell image analysis routines. Briefly, nuclear staining was used to identify single cells and the average cellular Aldefluor intensity in the DEAB inhibitor sample was determined. An intensity threshold was set at 6 standard deviations above DEAB average intensity. Next to the total number of cells, the number of cells above this intensity threshold was measured and the proportion of ALDH_high_ cells was calculated.

### TFC assay

Low adherent 384-well Scivax NanoCulture plates (Scivax Lifescience, renamed to ORGANOGENIX, Woburn, MA) or Greiner mircoplates with a cell-repellent surface (Greiner Bio-One, Kremsmünster, Austria) were preincubated with 20 µl serum-free Cancer Stem Cell Media Premium^TM^ (ProMab Biotechnologies, Richmond, CA, USA) at 37 °C and centrifuged to remove air bubbles. Cells were processed through a 40 µm filter and seeded at a limiting single-cell solution (100 or 200 cells/well) in 20 µl serum-free Cancer Stem Cell Media Premium^TM^ (ProMab Biotechnologies, Richmond, CA, USA) to the preincubated tumorsphere plates. Nuclei in Day0-control wells were stained with Hoechst 33,342 (1:5000, Life Technologies by Thermo Fisher, Waltham, MA, USA) and imaged using an ImageXpress Micro widefield imaging system (Molecular Devices, Sunnyvale, CA, USA) with a 10× air objective to determine exact seeding cell number using the MetaXpress software (Molecular Devices, Sunnyvale, CA, USA). After 7 days of growth, nuclei with Hoechst 33,342 (1:5000, Life Technologies by Thermo Fisher, Waltham, MA, USA) and dead cells with SytoxGreen (1:10,000, Life Technologies by Thermo Fisher, Waltham, MA, USA) were stained and images (bright field and fluorescent) were acquired using an ImageXpress Micro widefield imaging system (Molecular Devices, Sunnyvale, CA, USA) with a 10× air objective. MetaXpress software (Molecular Devices, Sunnyvale, CA, USA) and custom written image analysis routines were used to quantify tumorspheres. Briefly, Hoechst channel was used to detect nuclei and tumorspheres with a size between 50 and 250 µm and a shape factor of 0.5 were quantified. Tumorsphere count was normalized with initial seeding cell number to determine TFC. The main advantages of the developed TFC assay include that it is 384-well plate compatible, requires no tumorsphere transfer or matrix degradation for automated imaging, and shows no aggregation of cells at the well side after seeding.

### Separation of ALDH_high_ and ALDH_low_ cells by fluorescence-activated cell sorting (FACS)

To stain ALDH activity in SUM149 cells for subsequent FACS, the Aldefluor Kit (STEMCELL Technologies, Vancouver, Canada) was used according to the manufacturer's instructions. Briefly, 2 × 10^6^ cells/ml were incubated in Aldefluor assay buffer containing the activated Aldefluor reagent (1:200) at 37 °C for 45 min. For each experiment, some cells were additionally stained with the ALDH inhibitor DEAB (10 mM, STEMCELL Technologies, Vancouver, Canada) as negative control. After incubation cells were centrifuged and resuspended in fresh Aldefluor assay buffer containing 4,6-diamidino-2-phenylindole as viability stain. Until sorting, cells were kept on ice in the dark. To separate ALDH_high_ and ALDH_low_ cells, a FACS Aria machine (BD Biosciences, San Jose, CA, USA) was used. Gates were set using the viability stain and the DEAB inhibitor sample.

Purity of sorted populations was checked using double sorting of 10,000 ALDH_high_ and ALDH_low_ cells. The sorted ALDH_high_ populations contained >90% of ALDH_high_ cells and no ALDH_high_ cells were detected in the ALDH_low_ population.

### Treatment

For the small molecule screen, 20 µl culture medium containing either 80 nl compound of the ENZO Screen-Well ICCB Known Bioactives library (468 compounds, final compound dilution of 0.1–20 µM, depending on original stock concentration, Enzo Life Sciences (Farmingdale, NY, USA)) or 40 nl compound of the ENZO Screen-Well ICCB FDA-approved drug library (640 compounds, final compound dilution of 10 µM, Enzo Life Sciences (Farmingdale, NY, USA)) were added and incubated for 3 days at normal culture conditions (21% O_2_, 37 °C, 5% CO_2_). A 0.2% DMSO solution was used as solvent control.

Screening hits and further tool compounds were purchased from Sigma-Aldrich (St. Louis, MO, USA). All compounds were dissolved in DMSO (10 mM) and stored at −20 °C.

Normalization, quality control, and fitting curves for IC50 determination of tested compounds were done with Genedata Screener® for high-content screening and Genedata Condoseo modules (Genedata AG, Basel, Switzerland). In detail, wells with a reduced cell number >50% compared to DMSO control were masked and the proportion of ALDH_high_ cells was normalized to the DMSO control (0%) and the 10 µM DEAB (100%) control. For radiation, cells were seeded in 384-well imaging plates and radiated at different doses using a CellRad X-ray cell irradiator (Faxitron, Tucson, Arizona, USA).

The compound screen performance was characterized by a robust RZ’ factor of 0.3474 and a signal-to-background ratio of 2.22.

### Real-time quantitative PCR

Total RNA was isolated from cells or spheroids using RNeasy Plus Mini Kit (Qiagen, Hilden, Germany) and reverse-transcribed with the RevertAid H Minus First Strand cDNA Synthesis Kit (Thermo Fisher, Waltham, MA, USA) according to the manufacturer’s instructions. To measure the expression levels of target genes, sample concentrations were adjusted to 10 ng/µl cDNA and mixed with specific TaqMan Gene Expression Primer (Thermo Fisher, Waltham, MA, USA) and TaqMan Fast Advanced Master Mix (Thermo Fisher, Waltham, MA, USA). Real-time quantification was performed in quadruplicates on a MicroAmp optical 384-well reaction plate (Thermo Fisher, Waltham, MA, USA) using a 7900 PCR machine (Applied Biosystems). Relative mRNA levels were calculated to the geometric mean of reference gene RPL32 (encoding ribosomal protein L32).

#### TaqMan primers

Taqman primers used were: *RPL32* (ribosomal protein L32, Hs00851655_g1), *ALDH1A1* (aldehyde dehydrogenases 1A1, Hs00946916_m1), *ALDH1A2* (Hs00180254_m1), *ALDH1A3* (Hs00167476_m1), *ALDH1B1* (Hs00377718_m1), *ALDH1L2* (Hs00402876_m1), *ALDH2* (Hs01007998_m1), *ALDH3A1* (Hs00964880_m1), *ALDH3A2* (Hs00166066_m1), *ALDH7A1* (Hs00988965_m1), *ALDH8A1* (Hs00988965_m1), *SOX2* (SRY (sex determining region Y)-box 2, Hs01053049_s1), *KLF4* (Kruppel like factor 4, Hs00358836_m1), *POU5F1* (POU class 5 homeobox 1, Hs04260367_gH), and *PDE5A* (phosphodiesterase 5A, Hs00153649_m1).

### siRNA transfection

To generate gene knockdown cells, SUM149 cells were incubated with 10 nM siRNA and Lipofectamine RNAiMAX (Thermo Fisher, Waltham, MA, USA, 1:1000) lipid or control (lipid only) in 12- or 384-well plates and incubated for 3 days at 37 °C and 21% O_2_.

For ALDH activity staining, cells were transfected and stained in 384-well plates (for staining, see 'High-content-based Aldefluor assay and single-cell population analysis' section). *ALDH1A3* siRNA was used as positive siRNA control. The proportion of ALDH_high_ cells was normalized to the lipid only control (0%) and the *ALDH1A3* siRNA (100%) control.

For measuring the TFC, cells were transfected in 12-well plates for 3 days, detached using TrypLE Express (Gibco by Thermo Fisher, Waltham, MA, USA), and cell numbers were determined. Cells were seeded at a limiting single-cell solution (100 or 200 cells/well) in serum-free Cancer Stem Cell Media Premium^TM^ (ProMab Biotechnologies, Richmond, CA, USA) to Greiner mircoplates with a cell-repellent surface (Greiner Bio-One, Kremsmünster, Austria), (see 'TFC assay' section). For cell number check, nuclei in Day0-control wells were stained with Hoechst 33,342 (1:5000, Life Technologies by Thermo Fisher, Waltham, MA, USA) and imaged using an ImageXpress Micro widefield imaging system (Molecular Devices, Sunnyvale, CA, USA) with a 10× air objective to determine exact seeding cell number using the MetaXpress software (Molecular Devices, Sunnyvale, CA, USA). After 7 days of growth, nuclei with Hoechst 33,342 (1:5000, Life Technologies by Thermo Fisher, Waltham, MA, USA) and dead cells with SytoxGreen (1:10,000, Life Technologies by Thermo Fisher, Waltham, MA, USA) were stained, and images (bright-field and fluorescent) were acquired using an ImageXpress Micro widefield imaging system (Molecular Devices, Sunnyvale, CA, USA) with a 10× air objective. MetaXpress software (Molecular Devices, Sunnyvale, CA, USA) and custom written image analysis routines were used to quantify tumorspheres. Briefly, Hoechst channel was used to detect nuclei, and tumorspheres with a size between 50 and 250 µm and a shape factor of 0.5 were quantified. Tumorsphere count was normalized with initial seeding cell number to determine TFC.

#### siRNAs used

siRNAs used were *ALDH1A3* (s30, s32, Thermo Fisher, Waltham, MA, USA), *ALDH1A1* (1238, 1237, 1236, Thermo Fisher, Waltham, MA, USA), *PDE5A* (J-007667-14, J-007667-13, J-007667-12, GE Dharmacon, Lafayette, CO, USA), *PORCN* (35,006, 35,005, 35,004, Ambion by Thermo Fisher, Waltham, MA, USA), *TNKS1* (s16482, s16481, s16480, Ambion by Thermo Fisher, Waltham, MA, USA), *TNKS2* (s37281, s37280, s37279, Ambion by Thermo Fisher, Waltham, MA, USA), *FZD7* (15,843, 15,842, 15,841, Ambion by Thermo Fisher, Waltham, MA, USA), *LRP5* (8295, 8294, 8293, Ambion by Thermo Fisher, Waltham, MA, USA), *LRP6* (8290, 8291, 8292, Ambion by Thermo Fisher, Waltham, MA, USA), *TCF7L2* (s13882, s13881, s13880, Ambion by Thermo Fisher, Waltham, MA, USA), *TCF7L1* (37,905, 37,904, 37,906, Ambion by Thermo Fisher, Waltham, MA, USA), *TCF7* (13,879, 13,878, 13,877, Ambion by Thermo Fisher, Waltham, MA, USA), *LEF1* (27,616, 27,617, 27,618, Ambion by Thermo Fisher, Waltham, MA, USA), *SHH* (12,818, 12,817, 12,819, Ambion by Thermo Fisher, Waltham, MA, USA), *SMO* (13,165, 13,164, 13,166, Ambion by Thermo Fisher, Waltham, MA, USA), *GLI1* (s5816, s5815, s5814, Ambion by Thermo Fisher, Waltham, MA, USA), *GLI2* (s5817, s5818, s5819, Ambion by Thermo Fisher, Waltham, MA, USA), *ADAM10* (1004, 1005, 1006, Ambion by Thermo Fisher, Waltham, MA, USA), *NOTCH4* (9644, 9644, 224,126, Ambion by Thermo Fisher, Waltham, MA, USA), and *NOTCH1* (9634, 9633, 9635, Ambion by Thermo Fisher, Waltham, MA, USA).

## Electronic supplementary material

Supplementary Table 1

Supplementary Table 2

Supplementary Figure 1

Supplementary Figure 2

Supplementary Figure 3

Supplementary Figure 4

Supplementary Figure 5
