# Supplementary material for: PDE5 inhibition eliminates cancer stem cells via induction of PKA signaling
Source: Cell Death Dis. 2018 Feb 7;9(2):192. doi: 10.1038/s41419-017-0202-5 (PMC5833477; doi:10.1038/s41419-017-0202-5)
Supplement: Supplementary file 1 — Supplementary Table 1 [file 41419_2017_202_MOESM1_ESM.docx]

**Supplementary Table 1: Reduction of ALDH_high_ cells in SUM149 after Wnt-, Hh- or Notch-pathway inhibition.** SUM149 cells were treated for 72 h with either DMSO (cpd treatment)/lipid only (siRNA treatment) control or 10 µM compound/10 nM siRNA. Nuclei were stained with Hoechst and ALDH activity visualized by Aldefluor staining. The amount of ALDH_high_ cells was determined and normalized to controls. DEAB or *ALDH1A3* siRNA were used as inhibitor control to set intensity threshold of Aldefluor staining. Values were determined in ≥3 independent experiments. Coloring: Green = significant reduction ≤ -50 %, white= minimal reduction ≤10% % and > -50 %, red = no significant reduction.

| **Compound/ siRNA** | **Relative reduction ALDHhigh cells at 10 µM (cpd) / 10nM (siRNA), (%)** |
| --- | --- |
|  |  |
| **Wnt inhibitors** | |
| LGK-974 | -81.2 (± 9.91) |
| IWP-2 | 4.74 (± 0.55) |
| IWR-1 | -90.0 (± 4.5) |
| WIKI4 | -93.4 (± 5.27) |
| XAV-939 | 4.44 (± 1.18) |
| *PORCN* siRNA | -37.1 (± 1.94) |
| *TNKS1* siRNA | -22.8 (± 2.6) |
| *TNKS2* siRNA | 13.5 (± 17.6) |
| *FZD7* siRNA | -2.0 (± 1.75) |
| *LRP5* siRNA | -55.2 (± 6.02) |
| *LRP6* siRNA | -81.7 (± 1.52) |
| *TCF7L2* siRNA | -82.3 (± 0.12) |
| *TCF7L1* siRNA | -64.8 (± 0.11) |
| *TCF7* siRNA | -18.7 (± 2.67) |
| *LEF1* siRNA | -24.6(± 26.6) |
| **Hh inhibitors** | |
| Vismodegib (GDC-0449) | -32.9 (± 10.2) |
| Cyclopamine | 12.1 (± 3.29) |
| LDE225 (erismodegib) | -17.3 (± 11.6) |
| Jervine | 22.7 (± 13.53) |
| PF-04449913 | -35.3 (± 3.91) |
| *SHH* siRNA | -25.6 (± 8.7) |
| *SMO* siRNA | -54.6 (± 6.43) |
| *GLI1* siRNA | -46.8 (± 18.0) |
| *GLI2* siRNA | 15.9 (± 10.2) |
| **Notch inhibitors** | |
| MK-0752 | 6.6 (± 5.42) |
| RO-4929097 | 15.3 (± 3.59) |
| PF-3084014 | 3.2 (± 0.81) |
| DAPT | -4.5 (± 1.2) |
| *ADAM10* siRNA | -46.4 (± 2.4) |
| *NOTCH4* siRNA | -0.03 (± 4.67) |
| *NOTCH1* siRNA | -30.6 (± 1.54) |
