# Supplementary material for: PDE5 inhibition eliminates cancer stem cells via induction of PKA signaling
Source: Cell Death Dis. 2018 Feb 7;9(2):192. doi: 10.1038/s41419-017-0202-5 (PMC5833477; doi:10.1038/s41419-017-0202-5)
Supplement: Supplementary file 2 — Supplementary Table 2 [file 41419_2017_202_MOESM2_ESM.docx]

**Supplementary Table 2: IC50 generation in HCT116 cells of compounds that reduce ALDH_high_ cells in SUM149.** HCT116 cells were treated for 72 h and the amount of ALDH_high_ cells was determined and normalized to DMSO control. DEAB was used as inhibitor staining control to set intensity threshold of Aldefluor staining.

| **Compound** | **IC50 Reduction ALDH_high_ cells (M)** |
| --- | --- |
| **Wnt inhibitors** | |
| **LGK974** | not active |
| **IWR-1** | 1.99E-06 |
| **WIKI4** | 3.39E-06 |
| **Screening hits** | |
| **Celecoxib** | 1.36E-05 |
| **Cilostazol** | 2.63E-06 |
| **RHC-80267** | 1.74E-06 |
| **Dantrolene** | 8.10E-06 |
| **MY-5445** | 6.19E-06 |
| **Tyrphostin AG1478** | 1.23E-06 |
| **Xanthotoxin** | 7.59E-06 |
| **SB 203580** | NA |
| **PCA 4248** | 1.80E-06 |
| **Praziquantel** | 6.91E-06 |
