# Supplementary material for: PDE5 inhibition eliminates cancer stem cells via induction of PKA signaling
Source: Cell Death Dis. 2018 Feb 7;9(2):192. doi: 10.1038/s41419-017-0202-5 (PMC5833477; doi:10.1038/s41419-017-0202-5)
Supplement: Supplementary file 3 — Supplementary Figure 1 [file 41419_2017_202_MOESM3_ESM.pdf]

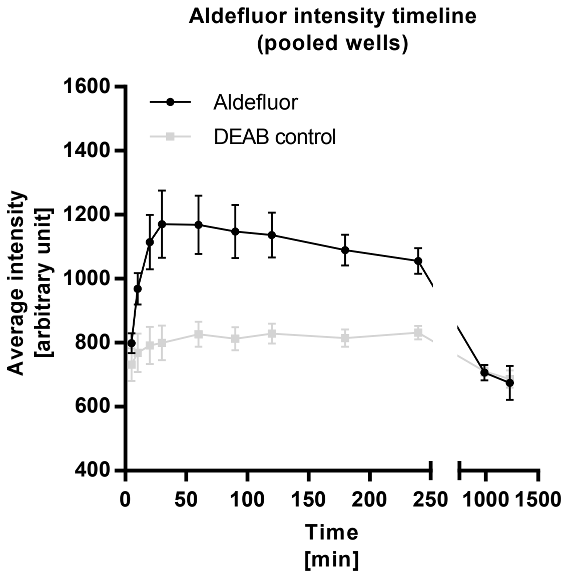

Supplementary figure 1: Signal stability of ALDH activity visualization by Aldefluor staining in a HCA based setup

SUM149 cells were plated in 384 well plates and stained with the Aldefluor reagent and the nuclear stain Hoechst. DEAB was used as inhibitor staining control. The signal intensity of ALDEFUOR was measured after various time points in multiple wells (n>10).
