# Supplementary material for: PDE5 inhibition eliminates cancer stem cells via induction of PKA signaling
Source: Cell Death Dis. 2018 Feb 7;9(2):192. doi: 10.1038/s41419-017-0202-5 (PMC5833477; doi:10.1038/s41419-017-0202-5)
Supplement: Supplementary file 4 — Supplementary Figure 2 [file 41419_2017_202_MOESM4_ESM.pdf]

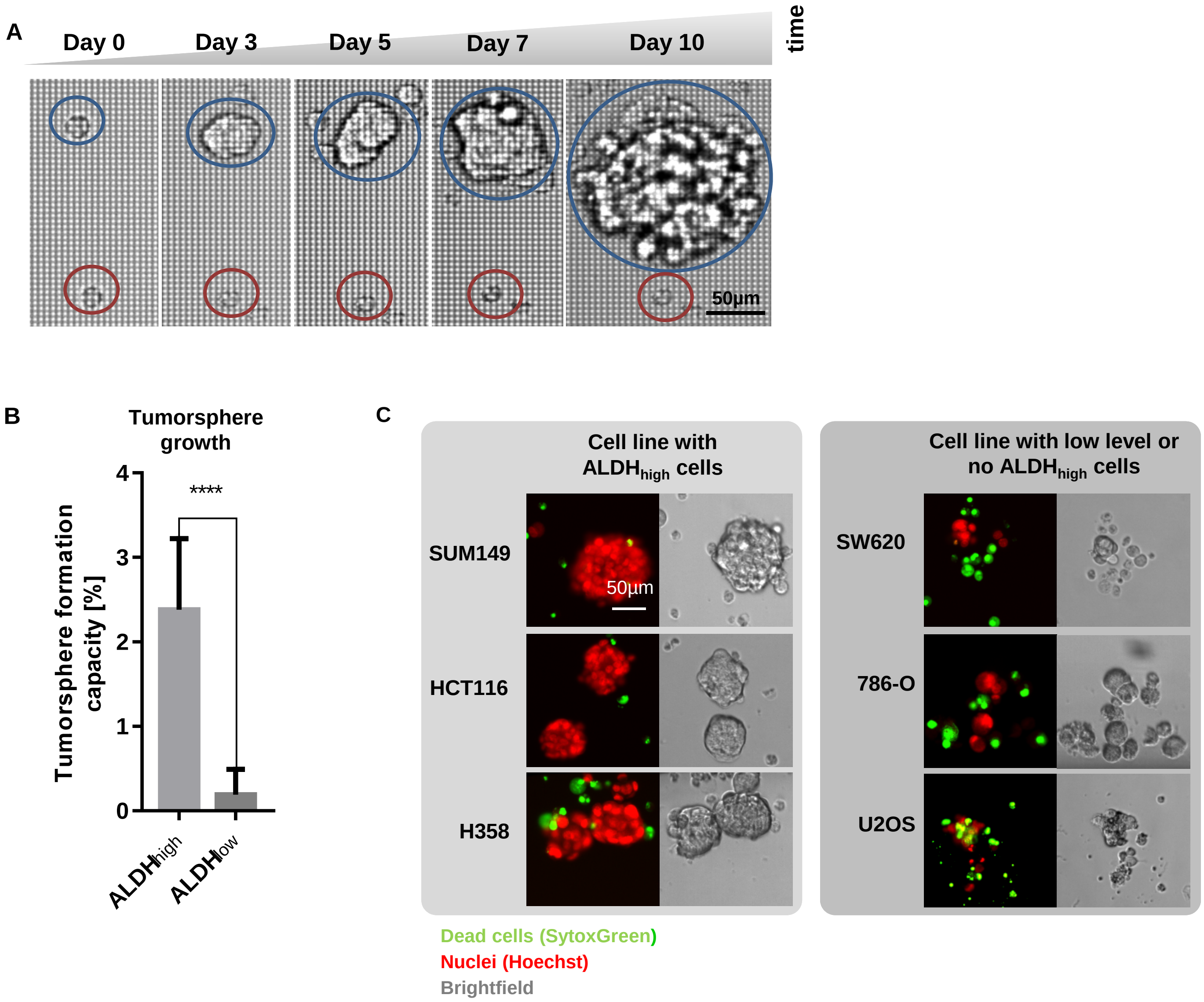

Supplementary figure 2: Monitoring tumorsphere formation from a single cell

A) A single cell suspension of SUM149 (100 cells per well) were plated in CSC medium into low adherent 384 well Scivax plates. Transmitted light images of the same site were acquired at multiple time points. Circled in red: cell showing no tumorsphere growth. Circled in blue: tumorigenic cell forming a tumorsphere. Scale bar 50 µm. B) SUM149 cells were sorted into stem-like $ALDH_{high}$ and non-stem-like $ALDH_{low}$ cells by FACS. Afterwards 200 cells of each population were plated in CSC medium into low adherent 384 well Scivax plates. After 7 days of growth, the number of tumorspheres was determined and normalized to the initial seeding cell number. Bars show mean with SD (n=3). **** p-value <0.0001. C) Tumorsphere growth of different cell lines. 100 cells of either SUM149, HCT116, H358, SW620, 786-O or U2OS were plated in CSC medium into 384 well Greiner mircoplates with a cell-repellent surface. At day 6, Nuclei were stained with Hoechst and dead cells with SytoxGreen. Brightfield images as well as fluorescent images were acquired. Scale bar 50 µm.
