# Supplementary material for: PDE5 inhibition eliminates cancer stem cells via induction of PKA signaling
Source: Cell Death Dis. 2018 Feb 7;9(2):192. doi: 10.1038/s41419-017-0202-5 (PMC5833477; doi:10.1038/s41419-017-0202-5)
Supplement: Supplementary file 5 — Supplementary Figure 3 [file 41419_2017_202_MOESM5_ESM.pdf]

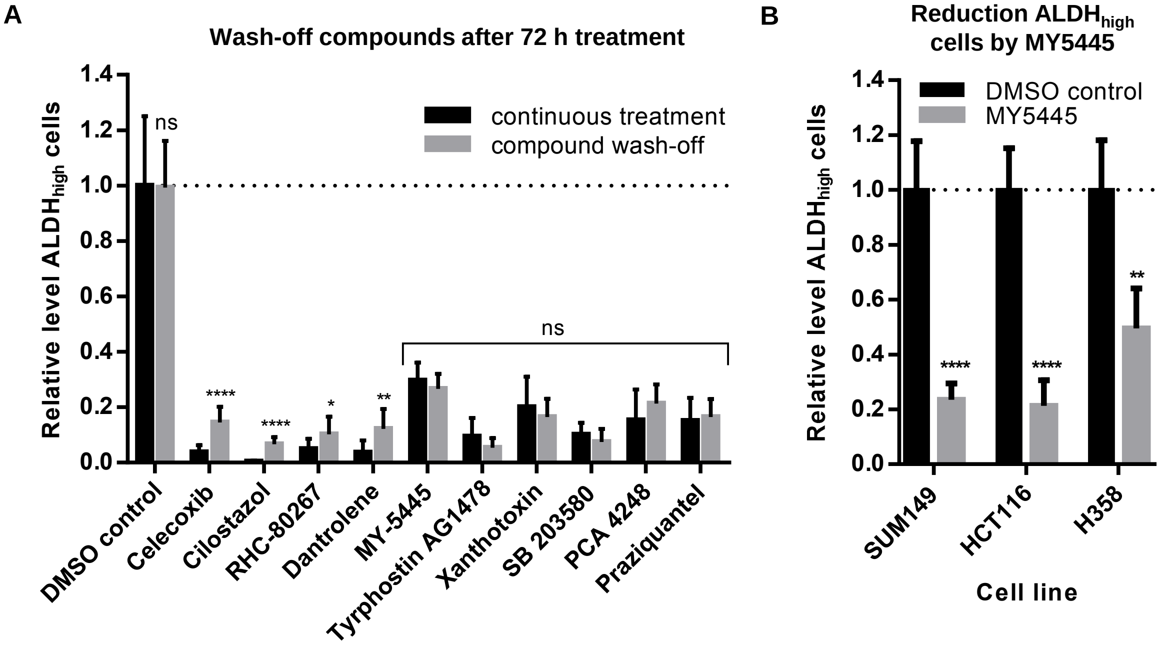

Supplementary figure 3: Reappearance of ALDH$_{high}$ cells 2 h after compound wash-off

A) SUM149 cells were treated for 72 h with either DMSO control or 10 µM compound. After 72 h medium was changed to wash-off compounds and cells were incubated for another 2 h in Aldefluor assay buffer (without compounds). The amount of ALDH$_{high}$ cells was determined and normalized to DMSO control. DEAB was used as inhibitor staining control to set intensity threshold. Bars show mean with SD (n=3). Significance was calculated for each compound comparing compound wash-off with no wash-off (continuous treatment). **** p-value <0.0001, *** p-value <0.001 ** p-value <0.01, * p-value <0.05, ns – not significant. B) SUM149, HCT116 and H358 cells were treated with either DMSO control or 10 µM MY5445. After 72 h, the amount of ALDH$_{high}$ cells was determined and normalized to DMSO control. DEAB was used as inhibitor staining control to set intensity threshold. Bars show mean with SD (n≥2). **** p-value <0.0001, ** p-value <0.01.
