# Supplementary material for: PDE5 inhibition eliminates cancer stem cells via induction of PKA signaling
Source: Cell Death Dis. 2018 Feb 7;9(2):192. doi: 10.1038/s41419-017-0202-5 (PMC5833477; doi:10.1038/s41419-017-0202-5)
Supplement: Supplementary file 6 — Supplementary Figure 4 [file 41419_2017_202_MOESM6_ESM.pdf]

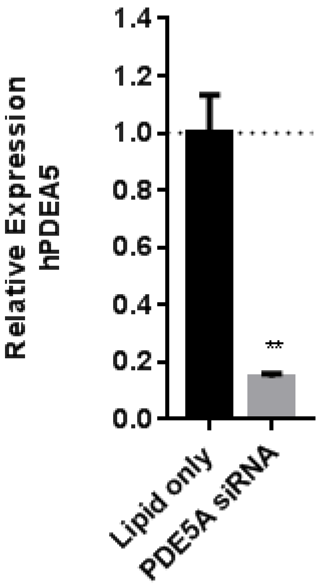

Supplementary figure 4: Gene expression analysis of PDE5A by RT-PCR in SUM149 cells treated for 72 h with either lipid only control or 10 nM PDE5A siRNA. hRP-L32 was used as reference gene and relative expression levels were normalized to lipid only control. Bars show mean with SD.
