# Supplementary material for: PDE5 inhibition eliminates cancer stem cells via induction of PKA signaling
Source: Cell Death Dis. 2018 Feb 7;9(2):192. doi: 10.1038/s41419-017-0202-5 (PMC5833477; doi:10.1038/s41419-017-0202-5)
Supplement: Supplementary file 7 — Supplementary Figure 5 [file 41419_2017_202_MOESM7_ESM.pdf]

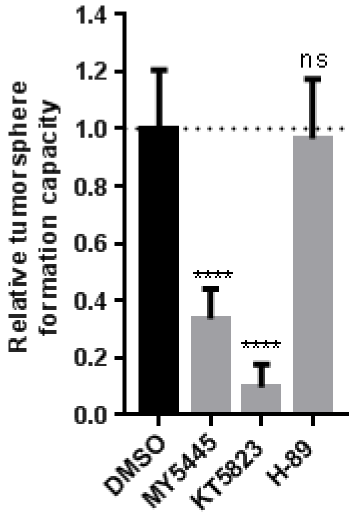

Supplementary figure 5: Reduction tumorsphere formation of SUM149 cells.

SUM149 cells were treated with either DMSO control, the PDE5 inhibitor MY5445 (10 µM), the PKG inhibitor KT5823 (10 µM) or the PKA inhibitor H-89 (5 µM) under anchorage-independent growth conditions (384 well tumorsphere plates). After 7 days of growth, the number of tumorspheres was determined and normalized to DMSO control. Bars show mean with SD. **** p-value <0.0001, ns-not significant.
